# Effectiveness of Hypoglossal-Facial Anastomosis in the Rehabilitation of Facial Paralysis Following Vestibular Schwannoma Surgery: A Systematic Review

**DOI:** 10.7759/cureus.57625

**Published:** 2024-04-04

**Authors:** Maiquel André Texeira Iora, Matheus Rodrigues Teixeira Netto, Camila Porto Cardoso, Pâmela Rossi dos Santos, Chariel Iserhardt Ciochetta, Jander Moreira Monteiro, Vagner Rodrigues, Gustavo Rassier Isolan, Joel Lavinsky

**Affiliations:** 1 Department of Emergency Medicine, Hospital São Camilo de Esteio, Porto Alegre, BRA; 2 Department of Internal Medicine, Hospital Dom João Becker, Gravatai, BRA; 3 College of Medicine, Universidade Federal de Ciências da Saúde de Porto Alegre, Porto Alegre, BRA; 4 Department of Internal Medicine, Hospital São Camilo de Esteio, Porto Alegre, BRA; 5 Department of Neurosurgery, The Center for Advanced Neurology and Neurosurgery (CEANNE), Porto Alegre, BRA; 6 Department of Otolaryngology - Head and Neck Surgery, Universidade Estadual de Campinas, Campinas, BRA; 7 Department of Morphological Sciences, Universidade Federal do Rio Grande do Sul, Porto Alegre, BRA

**Keywords:** hypoglossal-facial anastomosis, facial palsy, facial paralysis, skull base, vestibular schwannoma, facial nerve, hypoglossal nerve

## Abstract

The facial nerve plays a crucial role in facial expression and sensory functions, with irreversible injuries often demanding rehabilitation therapies, with hypoglossal-facial nerve anastomosis (HFA) being one of the treatment options. This systematic review assessed different HFA techniques for facial paralysis, particularly post vestibular schwannoma resection, focusing on effectiveness and associated morbidities. Fifteen studies, comprising a case series and a retrospective cohort, were analyzed. Techniques included end-to-end, split, side-to-side, end-to-side, and jump interpositional graft hypoglossal-facial anastomosis (JIGHFA). Positive outcomes were observed with end-to-end and side-to-side techniques, while the split technique and JIGHFA showed promise. Comparative analyses favored the ‘end-to-side’ approach. Shorter intervals between surgery and HFA correlated with improved outcomes. Methodological variations highlight the need for prospective studies with standardized methodologies for robust evidence and informed decision-making on optimal HFA techniques.

## Introduction and background

The facial nerve (FN) serves a crucial role in diverse physiological functions, encompassing motor coordination, sensory perception, and parasympathetic control. Its influence extends beyond the nuances of facial movement, impacting taste detection in the anterior ⅔ portion of the tongue and controlling the complex operations of the sublingual, submandibular, and lacrimal glands. This intricate neural network is, however, vulnerable to irreversible injuries, particularly in the context of surgical interventions or trauma to the skull base. The ramifications of such injuries transcend the physical domain, extending into the psychological sphere, where patients confront not only the tangible loss of facial mobility but also contend with the profound challenge of impaired social expression.

In the context of surgical interventions, the excision of tumors proximate to the FN, especially vestibular schwannomas (VS), may necessitate nerve resection [1]. Facial paralysis (FP) may result from these procedures, significantly impacting patients' quality of life and posing challenges in both functional and psychosocial aspects. Understanding the nuances of FP in this context is vital for tailoring effective rehabilitation strategies and managing the multifaceted consequences of FN damage. Hypoglossal-facial nerve anastomosis (HFA) becomes a treatment option for rehabilitating patients when a direct end-to-end anastomosis of the facial nerve itself is unattainable [2].

The evaluation of facial paralysis involves standardized measurements, such as the House-Brackmann (HB) scale, which quantifies the severity of facial nerve dysfunction. This scale provides a comprehensive assessment, ranging from normal (grade I) to total paralysis (grade VI), aiding in the subjective evaluation of treatment outcomes and the comparison of different HFA techniques [3].

The variety of techniques employed in HFA introduces nuances in the rehabilitation process for FP following VS resection. These techniques include end-to-end, split, side-to-side, end-to-side, side-to-end, and jump interpositional graft hypoglossal-facial anastomosis (JIGHFA). Each method presents distinct advantages and potential complications, shaping the landscape of facial rehabilitation.

This systematic review aims to scrutinize these diverse HFA techniques, evaluating their effectiveness and examining the associated morbidities to provide comprehensive insights into optimal treatment approaches for FP after VS resection.

## Review

Methods

Evidence Selection

The following search strategies for scientific articles were conducted on November 28, 2023, in the PubMed, Embase, and VHL databases, respectively: [PubMed] ("Neuroma, Acoustic"[Mesh] AND ("Facial Paralysis"[Mesh] OR "Hypoglossal Nerve"[Mesh] OR "Facial Nerve"[Mesh])) OR ("Facial Paralysis/rehabilitation"[Mesh] AND ("Hypoglossal Nerve"[Mesh] OR "Facial Nerve"[Mesh])); [Embase] 'neuroma, acoustic'/exp AND ('facial paralysis'/exp OR 'hypoglossal nerve'/exp OR 'facial nerve'/exp) OR ('facial paralysis/rehabilitation' AND ('hypoglossal nerve'/exp OR 'facial nerve'/exp)) AND [embase]/lim NOT ([embase]/lim AND [medline]/lim) NOT ('conference abstract'/it OR 'note'/it); and [VHL] Subject descriptor: ("Neuroma, Acoustic" AND ("Facial Paralysis" OR "Hypoglossal Nerve" OR "Facial Nerve")) OR ("Facial Paralysis/rehabilitation" AND ("Hypoglossal Nerve" OR "Facial Nerve")).

References were imported into the Rayyan (Qatar Computing Research Institute, Qatar) tool, and duplicates were removed [4]. The Population, Intervention, Comparison, Outcome (PICO) criteria were applied, encompassing patients with FP resulting from VS resection (P), who underwent rehabilitation using HFA (I), comparing various HFA techniques (C) to evaluate the effectiveness of these different HFA techniques in the rehabilitation of FP (O). Two independent reviewers conducted the initial analysis based on titles, abstracts, and, if necessary, full texts to exclude articles that met the predefined exclusion criteria. Excluded items encompassed review articles, videos, comments, articles lacking abstracts or full texts, studies involving animal models, studies unrelated to FP caused by VS surgery or its rehabilitation, and studies not utilizing the HFA technique for FP rehabilitation. Additionally, studies lacking separate analyses for HFA cases or VS cases, or studies not using the HB scale for outcome measurement were excluded. Any discrepancies were resolved through consensus. The reviewers subsequently reviewed all articles that remained after the initial exclusion step and reapplied the exclusion criteria.

Analysis of the Quality of Evidence

Analysis of the quality of evidence involved assessing the risk of bias in non-randomized studies [5]. The ROBINS-I tool was employed to analyze the risk of bias for each piece of evidence included in the review, considering aspects such as confounding, participant selection, intervention classification, deviations from intended interventions, missing data, outcome measurements, and selection of reported results [6].

For confounding, factors such as the consistency of the surgical team, age variations among patients, specification of the anastomosis type performed, the implementation of a rehabilitation protocol post-surgery, and whether primary or secondary data were utilized were evaluated. In the selection of study participants, scrutiny was given to sampling convenience, the interval between the VS resection and the rehabilitation surgery, the initial degree of paralysis, and the influence of VS size and FN status on participant selection. Concerning intervention classification, an assessment was made of the definition of each intervention and the timing of its execution. Evaluation of deviations from intended interventions considered whether deviations occurred and if they affected the relevant outcome. For missing data, an examination was made of participant data loss and its impact on results. Regarding outcome measurement, an evaluation was conducted on the objectivity of measuring outcomes, and for the selection of the reported result, analysis was carried out on the possible absence, omission, or manipulation of results.

Ultimately, based on the interpretation of bias risk for each item and its corresponding article, judgments were assigned according to Table 1 below.

**Table 1:** Risk of bias assessment

Criteria	Author (Year)														
	Ruschel et al. (2020) [7]	González-Darder et al. (2020) [1]	Dabiri et al. (2020) [8]	Przepiórka et al. (2020) [9]	Han et al. (2017) [10]	Yawn et al. (2016) [11]	Pardo-Maza et al. (2016) [2]	Zhang et al. (2015) [12]	Silva et al. (2012) [13]	Sood et al. (2000) [14]	Hammerschlag (1999) [15]	Samii and Matthies (1997) [16]	Kunihiro et al. (1996) [17]	Linnet and Madsen (1995) [18]	Arai et al. (1995) [19]
Bias due to confounding	Moderate	Moderate	Moderate	Low	Moderate	Moderate	Low	Low	Serious	Moderate	Low	Moderate	Moderate	Moderate	Moderate
Bias in selection of participants into the study	Low	Moderate	Moderate	Low	Moderate	Moderate	Moderate	Low	Moderate	Moderate	Low	Low	Moderate	Moderate	Moderate
Bias in classification of interventions	Low	Low	Low	Low	Low	Low	Low	Low	Low	Low	Low	Low	Low	Low	Low
Bias due to deviations from intended interventions	Low	Low	Low	Low	Low	Low	Low	Low	Low	Low	Low	Low	Low	Low	Low
Bias due to missing data	Low	Low	Low	Low	Low	Moderate	Low	Low	Low	Low	Low	Low	Low	Moderate	Low
Bias in measurement of the outcomes	Moderate	Low	Low	Low	Low	Serious	Low	Low	Low	Low	Low	Low	Low	Low	Low
Bias in selection of the reported result	Low	Low	Low	Serious	Low	Low	Serious	Low	Low	Low	Low	Low	Low	Low	Low
Overall risk of bias	Moderate	Moderate	Moderate	Serious	Moderate	Serious	Serious	Low	Serious	Moderate	Low	Moderate	Moderate	Moderate	Moderate

Data Extraction and Evidence Summarization

In each paper, the frequency of rehabilitated patients (those who presented HB grade III or lower) was extracted and categorized based on the surgical technique performed in each case. The effectiveness of HFA was assessed by calculating the ratio between the number of rehabilitated patients and the total number of patients who underwent surgery.

Results

The search resulted in 2,980 records, processed using the Rayyan tool [4] for screening. Initially, 1,220 duplicates were removed. Articles not published in English were also excluded, resulting in 1,438 records for screening. The first analysis aimed to exclude all articles that did not address HFA or VS in their abstracts. After screening, 71 reports were retrieved, and 56 were subsequently excluded due to outcomes different from those intended in this study, leaving 15 studies included in this systematic review, as presented in the preferred reporting items for systematic reviews and meta-analyses (PRISMA) flow diagram presented below.

**Figure 1:**
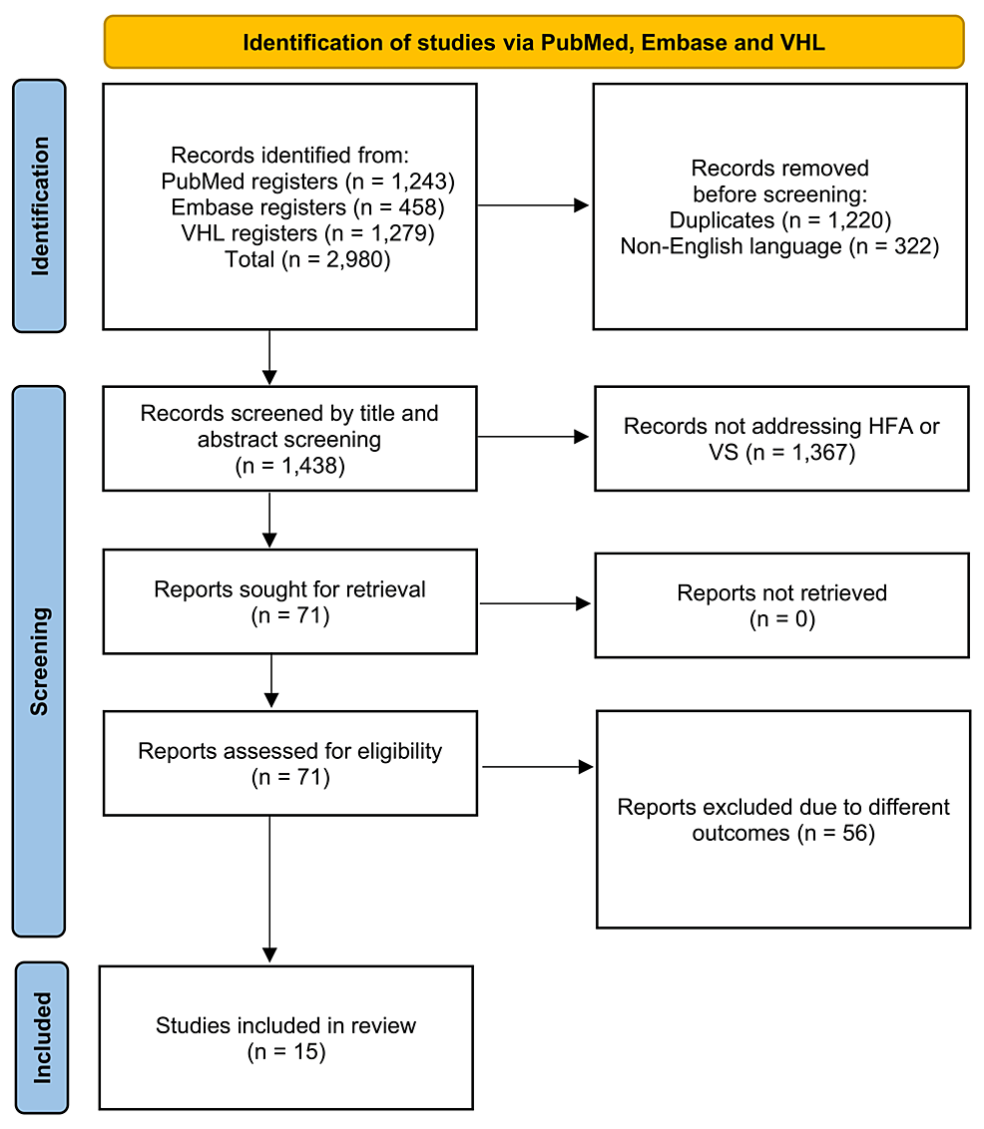
PRISMA 2020 flow diagram PRISMA: preferred reporting items for systematic reviews and meta-analyses

Upon analysis of the selected articles, two were classified as low risk of bias, nine as moderate risk of bias, and four as serious risk of bias, as shown in Table 2 below.

**Table 2:** Studies outcomes summary HFA: hypoglossal-facial anastomosis; JIGHFA: jump interpositional graft hypoglossal-facial anastomosis, VS: vestibular schwannoma; HB: House-Brackmann scale; N/A: not applicable; N/S: not specified

Item	Author (Year)																	
	Ruschel et al. (2020) [7]	González-Darder et al. (2020) [1]	Dabiri et al. (2020) [8]	Przepiórka et al. (2020) [9]		Han et al. (2017) [10]			Yawn et al. (2016) [11]	Pardo-Maza et al. (2016) [2]	Zhang et al. (2015) [12]	Silva et al. (2012) [13]	Sood et al. (2000) [14]	Hammerschlag (1999) [15]	Samii and Matthies (1997) [16]	Kunihiro et al. (1996) [17]	Linnet and Madsen (1995) [18]	Arai et al. (1995) [19]
HFA technique	Side-to-end	Side-to-end	End-to-side	Side-to-end	Hemi-end-to-end	End-to-end	End-to-side	Split	End-to-end (59), split (1)	End-to-end, hemi-end-to-end	Side-to-side	Unclear	Side-to-side	JIGHFA	Side-to-side	Side-to-end	End-to-end	Split
Number of patients	7	16	2	3	12	7	3	4	60	8	12	8	29	13	29	29	32	8
Bias risk	Moderate	Moderate	Moderate	Serious		Moderate			Serious	Serious	Low	Serious	Moderate	Low	Moderate	Moderate	Moderate	Moderate
Gender	3F 4M	12F 4M	1F 1M	N/S	N/S	4F 3M	3F	2F 2M	36F 24M	N/S	8M 4F	N/S	18F 11M	N/S	N/S	15M 14F	20F 12M	5F 3M
Age	51.4 (37-65)	50.8 (31-67)	49.5 (46-53)	N/S	N/S	38 (18-60)	28.3 (14-42)	37.5 (21-56)	49.3 (38-61)	N/S	39 (22-65)	N/S	55 (17-79)	40.1 (15-71)	N/S	48.7 (30-61)	46.5 (26-71)	56 (34-67)
Timeframe of intervention	4 d - 18 mo	7,8 mo (3-16)	12 mo	N/S	N/S	0 d - 6+ mo			14 d (8-365)	N/S	7.3 mo (1-24)	0d - 12 mo	15 mo (3-60)	6.5 mo (4-10)	7.8-14 mo	14-703 d	7 mo (0.4-22)	2.1 mo (1-6)
Follow-up period	36 mo	> 24 mo	18 mo	N/S	N/S	> 12 mo			> 12 mo	24 mo	29 mo (16-43)	39 mo (4-73)	42 mo (4-96)	28.8 mo (6-48)	>12 mo	~83 mo (15-192)	156 mo (24-288)	50.4 mo (36-63.6)
VS size	>3.5cm	9.2cc (3-25.1)	N/S	N/S	N/S	N/S	N/S	N/S	N/S	2.19cm (0.8-6)	N/S	4-6.1cm	N/S	3-7.5cm	N/S	N/S	N/S	N/S
HB grade pre-intervention	VI	VI	VI	N/S	VI	VI	VI	VI	V-VI	V-VI	V-VI	N/S	VI	VI	VI	VI	VI	V-VI
Number of intervention patients	7	16	2	3	12	7	3	4	60	8	12	8	29	13	29	29	32	8
Number of control patients	N/A	N/A	N/A	N/A	N/A	N/A	N/A	N/A	N/A	N/A	6	N/A	N/A	N/A	N/A	N/A	N/A	N/A
Number of rehabilitated intervention patients	7	16	2	2	11	5	1	3	43	7	9	3	19	10	23	7	8	8
Number of rehabilitated control patients	N/A	N/A	N/A	N/A	N/A	N/A	N/A	N/A	N/A	N/A	1	N/A	N/A	N/A	N/A	N/A	N/A	N/A
Intervention rehabilitation rate	100%	100%	100%	67%	92%	71%	33%	75%	72%	88%	75%	38%	66%	77%	79%	24%	25%	100%
Control rehabilitation rate	N/A	N/A	N/A	N/A	N/A	N/A	N/A	N/A	N/A	N/A	17%	N/A	N/A	N/A	N/A	N/A	N/A	N/A

The study conducted by Ruschel et al. (2020) comprises a preoperative and postoperative case series involving 12 patients with FP secondary to surgical procedures who underwent HFA using the side-to-end technique, with seven of them experiencing FP secondary to VS resection [7]. In addition to assessing the HB scale grades and electromyography, the patients were analyzed for the functions of their cranial nerves, including facial mimic, facial tonicity, tongue atrophy, and swallow disorders. Clinical evaluations were conducted at three, six, and 12 months, with an average follow-up of three years.

This study is considered to have a moderate risk of bias because it does not clarify whether a rehabilitation program was consistently applied to all patients and fails to stratify some results, such as the average paresis time and the average time of recovery, when considering other etiologies together. This limitation impedes the separate analysis of the outcomes of HFA due to VS resection. Additionally, the evaluation was deemed somewhat subjective.

A study conducted by González-Darder et al. (2020) is a pre-post study involving 16 patients who underwent HFA using the side-to-end technique following FP secondary to VS resection through the retrosigmoid approach [1]. All participants engaged in the same rehabilitation program and received specific clinical treatment. Motor recovery, facial movements during speech and eating, as well as tongue atrophy, were assessed every six months, with a minimum follow-up period of two years.

This study was deemed to have a moderate risk of bias because it did not specify how the patients included in the study were selected.

The case series conducted by Dabiri et al. (2020) assessed 10 patients with FP graded as VI due to proximal FN injury, with only two cases resulting from VS surgeries [8]. The patients underwent HFA using the end-to-side technique and were followed up for 18 months post-surgery. Notably, none of the patients exhibited tongue atrophy or reported any other side effects.

This study is characterized as a moderate risk of bias as it lacks clear information on the patient selection process and does not provide details about potential non-surgical co-interventions that could influence the success of patients' rehabilitation.

The case series by Przepiórka et al. (2020) assessed 29 patients who underwent surgery following VS removal, with different objectives such as revision, resection, or rehabilitation [9]. Among them, 15 patients underwent HFA using the side-to-end and hemi-end-to-end techniques.

Despite being executed with substantial methodological rigor, this study was categorized as having a serious risk of bias. This classification stems from the absence of detailed reporting on the specific demographic and clinical information of patients who underwent HFA, hindering the comprehensive evaluation of results and comparison with other studies.

Han et al. (2016) performed a before-and-after study involving 21 patients who underwent HFA following FP resulting from VS resection [10]. Among these patients, only 14 were included in the study. The performed techniques included end-to-end, end-to-side, and split methods, with evaluations focusing on the degrees of FP and tongue atrophy.

This study was categorized as having a moderate risk of bias due to the absence of specific criteria for patient selection. Additionally, the study did not provide information on whether participants engaged in any form of rehabilitation program, and potentially influencing the results, did not elucidate the reasons for the loss of follow-up for seven patients.

The study by Yawn et al. (2016) utilizes secondary data to establish a retrospective case series involving 60 patients who underwent HFA as part of FP rehabilitation surgery caused by VS resection [11]. In the majority of cases, the end-to-end technique was employed, except for 3% of cases where the anastomosis was partial. The degree of tongue atrophy was not evaluated.

This study has been designated as having a serious risk of bias, primarily due to the utilization of data collected from medical records, which raises concerns about the uniformity of outcome measurements across all patients. Additionally, the study lacks clarification regarding interventions concurrent with the surgical technique, such as rehabilitation programs.

The retrospective cohort study conducted by Pardo-Maza et al. (2016) does not specifically aim to assess the effectiveness of HFA but rather focuses on the evolution of 50 patients with FP secondary to VS resection [2]. Among them, only eight patients underwent HFA surgery, utilizing the end-to-end and hemi-end-to-end techniques. The study did not evaluate the degree of tongue atrophy.

This study has been categorized as having a serious risk of bias, primarily because it did not provide details about the characteristics of the sample of patients undergoing HFA. Furthermore, being a retrospective study that relies on secondary data, it raises concerns about potential biases inherent in such study designs.

The study by Zhang et al. (2015) is a non-randomized clinical trial involving 18 patients with FP after VS resection [12]. The intervention group comprised 12 patients who underwent HFA along with rehabilitation exercises, while the control group comprised six patients who performed only the exercises. The HFA was performed using the side-to-side technique. The evaluation encompassed facial rehabilitation, tongue movement, and electrophysiological study of facial muscles.

This study is regarded as having a low risk of bias, indicating that it exhibits a methodological approach without substantial biases that might affect the validity of the results.

The study conducted by Silva et al. (2012) is a retrospective case series that assessed 29 patients, primarily aiming to evaluate the prognosis of individuals after VS resection and not the effectiveness of HFA [13]. Among these patients, eight underwent HFA surgery. However, the study did not report the surgical technique performed.

This study has been categorized as having a serious risk of bias due to several factors. It did not provide details about the characteristics of patients undergoing HFA, failed to specify the surgical technique used, did not clarify whether there was co-intervention with rehabilitation programs, and was conducted as a retrospective study, introducing inherent limitations associated with such study designs.

The before-and-after study conducted by Sood et al. (2000) assessed 29 patients who underwent HFA following FP secondary to VS resection [14]. In addition to evaluating motor recovery using the HB scale, the study measured the benefit of surgery for patients using the Glasgow scale [20]. However, the study did not evaluate the degree of tongue atrophy.

This study is considered to have a moderate risk of bias because it lacks specificity regarding participant selection and does not report whether patients received any interventions beyond surgery, such as rehabilitation programs.

Hammerschlag (1999) conducted a before-and-after study in which 18 patients underwent HFA surgery, with 13 of them having FP caused by VS resection [15].

This study is considered to have a low risk of bias, as it does not exhibit significant methodological biases.

Samii and Matthies (1997) conducted a retrospective cohort study that assessed 1,000 patients who underwent VS resection. Among these, 29 individuals underwent HFA surgery for FP rehabilitation [16].

This study is categorized as having a moderate risk of bias due to certain factors. The authors did not explicitly specify whether the study is retrospective or prospective, or the HFA technique used, and there is a partial presentation of the characteristics of patients undergoing HFA.

The before-and-after study conducted by Kunihiro et al. (1996) analyzed 29 cases of HFA after VS resection. According to the references in the text, the technique used was the side-to-end technique. The study did not include an assessment of tongue atrophy [17].

This study is characterized as having a moderate risk of bias due to several factors. It does not specify whether patients received co-interventions such as rehabilitation programs, and although it suggests the surgical technique used, it does not fully clarify the procedural details.

The retrospective study by Linnet and Madsen (1995) involved the evaluation of 32 patients who underwent HFA surgery following FP secondary to VS resection [18]. The surgical technique adopted for the procedures was end-to-end.

This study is categorized as having a serious risk of bias due to several critical factors. Primarily, it does not clearly state whether the measurement of FP degree was conducted through face-to-face medical assessment or via a questionnaire. Additionally, the study presents a significant loss of patients, lacks clarification on the presence of any co-intervention that might have introduced confusion in the analysis of outcomes and does not specify the criteria for patient selection.

The case series presented by Arai et al. (1995) assessed eight patients who underwent HFA surgery using the split technique following FP caused by VS surgery [19].

This study is categorized as having a moderate risk of bias due to a lack of clarity about how patients were selected and a deficiency of details regarding co-interventions.

Discussion

This systematic review included 15 studies, consisting of 14 case series and one retrospective cohort, examining the effectiveness of HFA in individuals with FP after VS resection. Different techniques were employed for the anastomosis of the XII-VII nerves in the studies: three studies utilized the "end-to-end" technique, two used the "side-to-end" technique, one applied the "split" technique, one used the "end-to-side" technique, three employed the "side-to-side" technique, one used the "graft" technique, two compared different techniques, and one study did not specify the analyzed technique.

End-to-End

The classic end-to-end anastomosis technique between the hypoglossal and facial nerves, initially described by Körte in 1903, has been widely utilized for treating FP [21,22]. All five studies reported positive outcomes in terms of facial movement recovery, as assessed by the HB scale, following the HFA procedure [3,14]. Among these, two studies indicated issues related to tongue atrophy and mobility [14,17], while another study observed ocular dysfunctions such as dry eyes and hyperemia, necessitating tarsorrhaphy in some patients [17].

Despite variations in the time frame between VS resection and HFA, all articles highlighted improved outcomes when the anastomosis was performed within two years of VS removal [11,14,16-18]. This timeframe aligns with findings from Conley and Baker (1979), who analyzed 137 patients with FP, including 16 resulting from VS removal, treated with HFA [23]. They proposed that a shorter interval for performing the anastomosis is associated with a lower incidence of mastication, swallowing, and speech disorders.

Split/Hemi-Hypoglossal Facial

The study conducted by Arai et al. (1995) explored the application of the hypoglossal split technique for anastomosis with the facial nerve in the rehabilitation of FP following VS resection [19]. The results demonstrated that all eight patients exhibited progress on the HB scale, transitioning from grades V and VI to grade III after the intervention in a period of 12 months. Notably, mild or moderate hemi-atrophy of the tongue, which also resolved within the one-year period, was observed. Strong mass movements were noted in two patients during eating or talking, but neither patient complained.

Surgical procedures for facial resuscitation were conducted within six months after VS resection, suggesting a correlation with other findings that indicate better outcomes for interventions in nerves with less injury time, although this consensus is not universal [17,23,24].

Side-to-Side

The study by Zhang et al. (2015) introduces a novel technique for surgically rehabilitating facial nerve function after VS surgery [12]. This method involves the anastomosis of a pre-degenerated nerve graft, connecting proximally to the hypoglossal nerve and distally to the injured facial nerve. In this clinical trial, 10 out of the 12 participants in the intervention group were successfully rehabilitated during the follow-up period. Among them, five achieved HB grade II, while rehabilitation occurred in only one of the six participants in the control group, indicating the effectiveness of the technique.

The authors attribute the lack of success in rehabilitating the remaining two patients to the duration of paralysis exceeding one year. Importantly, no participant experienced any side effects related to hypoglossal nerve injury. This technique is grounded in the regenerative response of Schwann cells after injury [25], a process still in an experimental phase. Nevertheless, advancements in recent years have shown promise for its potential clinical application [26].

End-to-End x End-to-Side x Split

The study by Han et al. (2016) undertook a comparison of outcomes associated with three HFA techniques performed after VS resection [10]. In this case series, 14 patients were evaluated, with seven undergoing the "end-to-end" technique, three the "end-to-side" technique, and four the "split" technique. The results indicated favorable facial outcomes for all patients, regardless of the technique used. Regarding tongue morbidity, more than half of the patients achieved satisfactory results.

However, when comparing the techniques, patients who underwent the "end-to-side" approach achieved 100% favorable outcomes, while those who underwent the "split" technique had 75% favorable outcomes, and those who underwent the ‘end-to-end’ approach had approximately 28%. Statistical analysis demonstrated no significant differences in facial and tongue outcomes among the techniques, tumor size, or time between VS resection and HFA.

Despite this, the authors prefer the "split" technique when feasible, as it consistently yields positive results in facial mobility and tongue tropism, aligning with findings by Arai et al. (1995) [19]. Regarding time, better outcomes were observed in patients with an interval of up to 12 months between surgery and HFA, in agreement with suggestions from other authors [17,23,24].

Side-to-End

Ruschel et al. (2020) analyzed the 'side-to-end' HFA technique to assess seven patients with FP secondary to VS resection [7]. All patients initiated with grade VI on the HB scale, and all exhibited positive outcomes. One achieved grade II, five advanced to grade III, and one progressed to grade IV. The timeframe of intervention varied from four days to 18 months, and improvement occurred between three and six months, with an average follow-up of three years. The study concluded that a shorter mean duration of FP until intervention correlated with a swifter recovery. Importantly, no instances of lingual atrophy or swallowing disorders were observed after the intervention.

González-Darder et al. (2020) analyzed the impact of the "side-to-end" HFA technique for facial nerve resuscitation in 16 patients with irreversible FP after VS removal [1]. The results indicated improvement in facial movements for all patients, with 15 achieving grade III and one achieving grade IV on the HB scale. Notably, no occurrences of tongue atrophy or synkinesis were observed. The interval between VS removal surgery and the anastomosis averaged approximately eight months, with a range of three to 16 months.

Dabiri et al. (2020) studied 10 patients with FP, of which two were secondary to VS resection [8]. The findings showed that these patients achieved a favorable facial mimic response (grade III on the HB scale) without speech or swallowing sequelae due to tongue atrophy. The conclusions drawn from these studies align with other works in the literature, collectively demonstrating that the "side-to-end" HFA technique can be a favorable option for improving paralysis and reducing morbidities [27-29].

Jump Interpositional Graft Hypoglossal-Facial Anastomosis x End-to-End

The study conducted by Hammerschlag (1999) compared the outcomes of the "end-to-end" HFA technique with the "jump interpositional graft hypoglossal-facial anastomosis (JIGHFA)" technique in the rehabilitation of FP after surgery [15]. The JIGHFA group, consisting of 18 patients (13 with paralysis due to VS resection and 12 treated with electromyography), was compared to a group that received end-to-end treatment. This comparison group comprised 30 patients from a case series by Brudny et al. (1988) and 22 patients who responded to an anonymous questionnaire about their perception after rehabilitation surgery using the same technique associated with electromyography [30].

Results showed that both the JIGHFA group and Brudny's series achieved levels between II and IV on the HB scale. Among the patients subjectively evaluated, 15 had good results (grades between II and IV on the HB scale), while seven had undesirable results (level V on the HB scale).

In terms of morbidities, ophthalmic complaints were more prevalent in the classic HFA group, including drooping eyebrows and eyelids, synkinesis when closing the eyes, impaired vision, and chronic tearing. It's noteworthy that 17 patients in the JIGHFA group and the classic HFA group underwent eye surgery with golden-weight eyelid implantation and canthoplasty, respectively. Hypertonia was more prevalent among patients undergoing classic HFA, while synkinesis was more common in the JIGHFA group.

Regarding tongue morbidity, such as speech and swallowing issues, no problems were found in patients in the JIGHFA group, whereas 10 out of 22 patients undergoing classic HFA reported such problems. The study's findings are consistent with other literature assessing grafts for HFA, demonstrating a positive response to facial mimicry recovery with the presence of synkinesia and few or no issues related to tongue tropism [28,31].

End-to-End and Hemi-End-to-End

Pardo-Maza et al. (2016) reported on a retrospective cohort of 50 patients who underwent facial nerve resuscitation procedures [2]. Among the various techniques presented, only eight patients were treated with HFA, with four undergoing the "end-to-end" technique and four undergoing the "hemi-end-to-end" technique. The results are not presented separately, preventing an analysis of the effectiveness of the described approaches. However, the study introduces an algorithm for selecting the performance of HFA, taking into account factors such as nerve integrity verified by electrostimulation, the possibility of spontaneous recovery, and the availability of sections of the sectioned nerve.

Silva et al. (2012) conducted a retrospective study on 29 patients who underwent surgery for the resection of giant neuromas, and eight of these patients required HFA [13]. However, the authors do not specify which technique was used, impeding the analysis for the present study.

## Conclusions

The objective of this systematic review was to analyze the effectiveness and application of different techniques of hypoglossal-facial anastomosis (HFA) and highlight the varying levels of rehabilitation of facial functions and the morbidities generated by the procedure. The goal is to facilitate individual decision-making, supported by scientific evidence, regarding which techniques offer the best benefit to the patient with the least possible harm.

The available and analyzed literature, although diverse, comprises heterogeneous studies, making it challenging to perform an effective direct comparison of the techniques. Nevertheless, it is apparent that techniques involving a lower degree of damage to the nerves for anastomosis tend to yield better outcomes in the recovery of facial movements. This is attributed to fewer morbidities caused to patients who already have those inherent to their condition. Another contributing factor to improved clinical outcomes in functional rehabilitation is a shorter time between facial nerve injury and the surgery in which the anastomosis is performed, preferably within 12 months.

It is crucial to note that, given the diverse methodological limitations across the studies and variations in the experience levels of the centers performing the exemplified techniques, the results of this work cannot be generalized to all situations.

Therefore, there is a need for new prospective studies that compare the different techniques in similar groups of patients to obtain more robust evidence regarding the optimal approach for performing HFA.
